# Supplementary material for: The Effect of Low Irradiance on Leaf Nitrogen Allocation and Mesophyll Conductance to CO2 in Seedlings of Four Tree Species in Subtropical China
Source: Plants (Basel). 2021 Oct 18;10(10):2213. doi: 10.3390/plants10102213 (PMC8540425; doi:10.3390/plants10102213)
Supplement: Supplementary file 1 [file plants-10-02213-s001.zip › plants-1377753-supplementary.pdf]

The Effect of Low Irradiance on Leaf Nitrogen Allocation and Mesophyll Conductance to CO_2 in Seedlings of Four Tree Species in Subtropical China

Jingchao Tang [1,2], Baodi Sun [1], Ruimei Cheng [2,3], Zuomin Shi [2,3,4,*], Da Luo [2], Shirong Liu [2] and Mauro Centritto [4]

[1] School of Environmental and Municipal Engineering, Qingdao University of Technology, 266525 Qingdao, China; tjc@qut.edu.cn (J.T.); sunbaodi0927@126.com (B.S.)

[2] Key Laboratory of Forest Ecology and Environment of National Forestry and Grassland Administration, Research Institute of Forest Ecology, Environment and Protection, Chinese Academy of Forestry, 100091 Beijing, China; chengrm@caf.ac.cn (R.C.); luoda2010@163.com (D.L.); liusr@caf.ac.cn (S.L.)

[3] Co-Innovation Center for Sustainable Forestry in Southern China, Nanjing Forestry University, 210037 Nanjing, China

[4] Institute for Sustainable Pant Protection, National Research Council of Italy, Strada delle Cacce 73, 10135 Torino, Italy; mauro.centritto@cnr.it

* Correspondence: Correspondence: shizm@caf.ac.cn; Tel.: +86-010-62888308

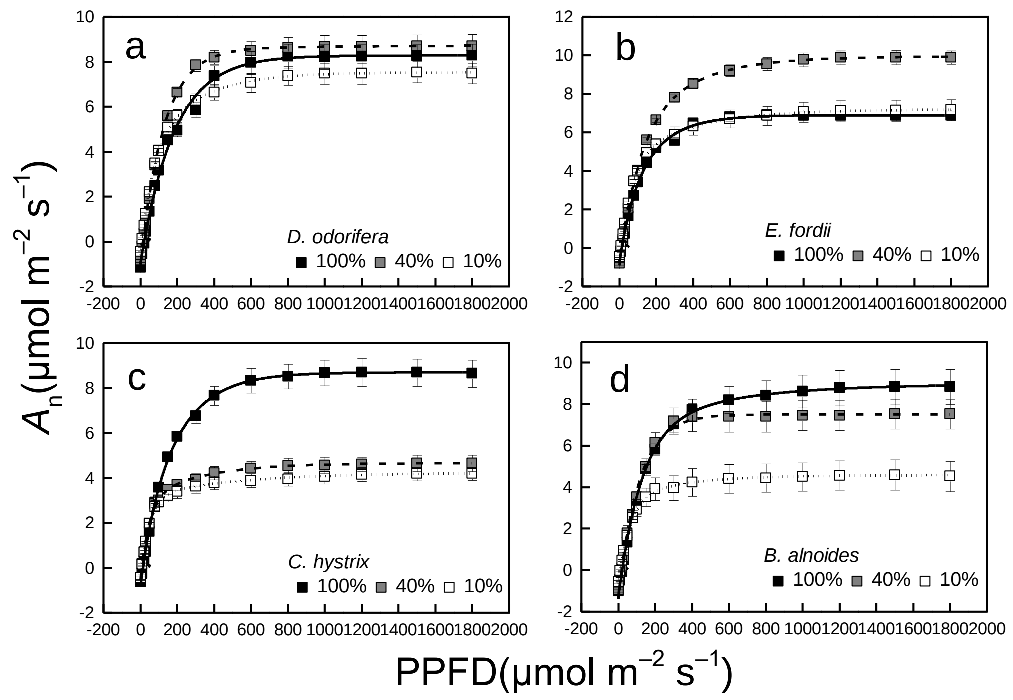

Figure S1. A_n-PPFD curves in *Dalbergia odorifera, Erythrophleum fordii, Betula alnoides,* and *Castanopsis hystrix* grown under three different irradiance treatments. Data are means of seven plants per treatment ± SE.

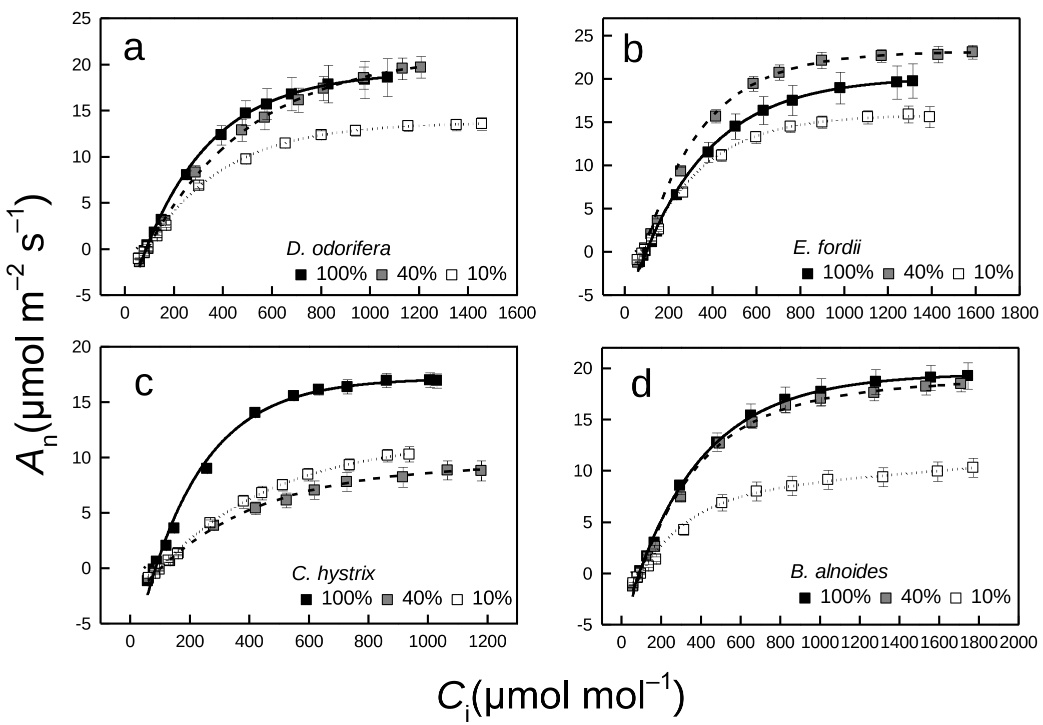

Figure S2. A_n-C_i curves in *Dalbergia odorifera*, *Erythrophleum fordii*, *Betula alnoides*, and *Castanopsis hystrix* grown under three different irradiance treatments. Data are means of seven plants per treatment ± SE.

Table S1 Mesophyll conductance (g_m), and CO_2 concentration at carboxylation site (C_c) calculated by three methods in four species seedling leaves under different irradiance treatments. Data are means of seven plants per treatment ± SE. Lower case letters indicate significant difference at 0.05 levels among the irradiance treatments, whereas capital letters indicate significant difference at 0.05 levels among species under same irradiance treatment. *F*-ratios with statistically significant values denoted by $^{*}P < 0.05$, $^{**}P < 0.01$, $^{***}P < 0.001$ among irradiance treatment.

Tree species	N treatments	g_m(molCO_2 m^{-2} s^{-1}) Harley	g_m(molCO_2 m^{-2} s^{-1})Ethier	g_m(molCO_2 m^{-2} s^{-1}) Gu	C_c(μmol mol^{-1}) Harley	C_c(μmol mol^{-1}) Ethier	C_c(μmol mol^{-1}) Gu
D. odorifera	HL	0.114±0.013^{aA}	0.140±0.011^{aA}	0.159±0.006^{aA}	178.394±7.837^{bB}	192.993±7.111^{bB}	200.924±6.715^{bA}
	ML	0.101±0.006^{aA}	0.107±0.006^{bA}	0.115±0.006^{bA}	204.861±9.132^{abA}	209.466±8.782^{abA}	215.546±9.468^{abA}
	LL	0.097±0.006^{aA}	0.101±0.010^{bA}	0.102±0.007^{bA}	229.974±5.705^{aA}	230.803±7.631^{aA}	233.247±6.121^{aA}
	F	0.919*	5.326*	24.179***	11.254**	5.799*	4.565*
E. fordii	HL	0.068±0.007^{bB}	0.066±0.007^{bC}	0.063±0.007^{aB}	136.804±5.186^{bC}	133.559±7.949^{bC}	127.420±9.562^{bB}
	ML	0.090±0.005^{aA}	0.097±0.003^{aA}	0.102±0.004^{bA}	149.830±2.997^{abB}	158.317±4.972^{aB}	162.294±5.094^{aB}
	LL	0.069±0.005^{bB}	0.076±0.005^{bAB}	0.077±0.004^{aB}	165.191±5.735^{aB}	174.213±3.991^{aB}	175.534±4.659^{aB}
	F	4.389*	9.575**	14.780***	8.809**	12.128***	13.324***
C. hystrix	HL	0.109±0.006^{aAB}	0.099±0.004^{aBC}	0.087±0.008^{aB}	172.173±6.100^{bB}	173.927±3.732^{aB}	157.856±9.558^{bB}
	ML	0.051±0.006^{bB}	0.054±0.005^{bB}	0.055±0.006^{bB}	203.053±3.562^{aA}	193.010±7.087^{aA}	193.255±7.793^{aAB}
	LL	0.050±0.006^{bB}	0.052±0.005^{bB}	0.057±0.005^{bB}	182.809±5.973^{bB}	184.806±5.946^{aB}	191.789±7.410^{aB}
	F	33.111***	30.453***	7.402**	8.626**	2.763	5.813*
B. alnoides	HL	0.136±0.013^{aA}	0.129±0.011^{aAB}	0.138±0.016^{aA}	228.781±8.439^{aA}	224.911±9.124^{aA}	225.576±12.271^{aA}
	ML	0.101±0.010^{aA}	0.103±0.008^{aA}	0.108±0.009^{aA}	220.277±13.165^{aA}	222.796±11.669^{aA}	225.750±11.857^{aA}
	LL	0.053±0.009^{bB}	0.056±0.009^{bB}	0.059±0.008^{bB}	227.034±9.417^{aA}	231.153±9.229^{aA}	238.020±9.285^{aA}
	F	15.102***	15.266***	12.250***	0.182	0.186	0.405

Table S2 Quantity of leaf N per area allocated to Rubisco (Q_{Rarea}), bioenergetics (Q_{Barea}), light-harvesting components (Q_{Larea}), photosynthetic apparatus (Q_{Parea}), cell wall (Q_{CWarea}), and other parts ($Q_{Other-area}$) in four species seedling leaves under different irradiance treatments. Data are means of seven plants per treatment ± SE. Lower case letters indicate significant difference at 0.05 levels among the irradiance treatments, whereas capital letters indicate significant difference at 0.05 levels among species under same irradiance treatment. *F*-ratios with statistically significant values denoted by $^{*}P < 0.05$, $^{**}P < 0.01$, $^{***}P < 0.001$ among irradiance treatment.

Tree species	N treatments	Q_{Rarea}(g·m-2)	Q_{Barea}(g·m^{-2})	Q_{Larea}(g·m^{-2})	Q_{Parea}(g·m^{-2})	Q_{CWarea}(g·m^{-2})	$Q_{Other-area}$(g·m^{-2})
D. odorifera	HL	$0.288±0.012^{abAB}$	$0.064±0.004^{aB}$	$0.224±0.011^{aA}$	$0.577±0.019^{aA}$	$0.150±0.015^{B}$	$1.459±0.114^{aA}$
	ML	$0.324±0.028^{aB}$	$0.076±0.004^{aB}$	$0.213±0.004^{aA}$	$0.614±0.031^{aB}$	$0.107±0.010^{bC}$	$0.894±0.043^{bA}$
	LL	$0.232±0.011^{bC}$	0.052±0.002bB	0.176±0.004bBC	0.459±0.014bB	0.059±0.004cB	0.449±0.036cB
	F	6.189^{**}	12.718^{***}	13.703^{***}	12.575^{***}	18.036^{***}	47.921^{***}
E. fordii	HL	$0.330±0.029^{bA}$	$0.085±0.008^{bA}$	$0.117±0.017^{bB}$	$0.532±0.042^{bAB}$	$0.103±0.005^{aB}$	$1.372±0.099^{aA}$
	ML	$0.469±0.019^{aA}$	$0.115±0.005^{aA}$	$0.225±0.009^{aA}$	$0.808±0.013^{aA}$	$0.066±0.001^{bD}$	$0.875±0.022^{bA}$
	LL	$0.317±0.021^{bA}$	$0.064±0.002^{cA}$	$0.234±0.016^{aA}$	$0.614±0.033^{bA}$	$0.060±0.003^{bB}$	$0.883±0.028^{bA}$
	F	13.144^{***}	21.504^{***}	19.625^{***}	20.426^{***}	41.742^{***}	22.028^{***}
C. hystrix	HL	$0.308±0.010^{aAB}$	$0.069±0.002^{aAB}$	$0.073±0.008^{bB}$	$0.450±0.015^{aBC}$	$0.273±0.013^{bA}$	$0.299±0.023^{aB}$
	ML	$0.170±0.008^{bC}$	$0.036±0.003^{bC}$	$0.096±0.009^{abB}$	$0.302±0.013^{cD}$	$0.329±0.007^{aA}$	$0.123±0.055^{bB}$
	LL	$0.195±0.009^{bBC}$	$0.043±0.003^{bBC}$	$0.131±0.015^{aBC}$	$0.369±0.022^{bBC}$	$0.267±0.018^{bA}$	$0.153±0.027^{bC}$
	F	71.560^{***}	48.291^{***}	7.117^{**}	19.017^{***}	6.431^{**}	6.192^{**}
B. alnoides	HL	$0.250±0.006^{aB}$	$0.064±0.004^{aB}$	$0.116±0.009^{aB}$	$0.430±0.011^{aC}$	$0.230±0.029^{aA}$	$0.372±0.068^{aB}$
	ML	$0.273±0.015^{aB}$	$0.067±0.004^{aB}$	$0.090±0.007^{aB}$	$0.430±0.024^{aC}$	$0.150±0.016^{bB}$	$0.173±0.028^{bB}$
	LL	$0.157±0.016^{bB}$	$0.035±0.003^{bC}$	$0.098±0.007^{aC}$	$0.291±0.023^{bC}$	$0.085±0.011^{cB}$	$0.184±0.015^{bC}$
	F	20.767^{***}	21.539^{***}	2.781	15.374^{***}	12.978^{***}	6.674^{**}

Table S3 Quantity of leaf N per mass allocated to Rubisco (Q_{Rmass}), bioenergetics (Q_{Bmass}), light-harvesting components (Q_{Lmass}), photosynthetic apparatus (Q_{Pmass}), cell wall (Q_{CWmass}), and other parts ($Q_{Other-mass}$) in four species seedling leaves under different irradiance treatments. Data are means of seven plants per treatment ± SE. Lower case letters indicate significant difference at 0.05 levels among the irradiance treatments, whereas capital letters indicate significant difference at 0.05 levels among species under same irradiance treatment. *F*-ratios with statistically significant values denoted by $^{*}P < 0.05$, $^{**}P < 0.01$, $^{***}P < 0.001$ among irradiance treatment.

Tree species	N treatments	Q_{Rmass}(mg·g^{-1})	Q_{Bmass}(mg·g^{-1})	Q_{Lmass}(mg·g^{-1})	Q_{Pmass}(mg·g^{-1})	Q_{CWmass}(mg·g^{-1})	$Q_{Other-mass}$(mg·g^{-1})
D. odorifera	HL	4.241±0.236^{bA}	0.940±0.033^{cAB}	3.316±0.253^{cA}	8.498±0.457^{cA}	2.159±0.156^{aB}	21.045±0.857^{aA}
	ML	6.293±0.608^{bB}	1.474±0.097^{bB}	4.121±0.065^{bA}	11.888±0.739^{bB}	2.067±0.177^{aC}	17.219±0.646^{bA}
	LL	7.950±0.464^{aA}	1.767±0.088^{aA}	6.007±0.084^{aA}	15.724±0.586^{aA}	2.008±0.148^{aC}	15.276±1.107^{bB}
	F	16.151***	28.712***	76.204***	35.719***	0.222	10.867**
E. fordii	HL	4.611±0.391^{cA}	1.192±0.104^{bA}	1.639±0.239^{cB}	7.442±0.559^{bAB}	1.442±0.060^{aC}	19.202±1.265^{abA}
	ML	8.836±0.357^{aA}	2.162±0.101^{aA}	4.245±0.188^{bA}	15.243±0.291^{aA}	1.246±0.015^{bD}	16.485±0.373^{bA}
	LL	7.184±0.488^{bA}	1.452±0.089^{bB}	5.295±0.338^{aA}	13.932±0.768^{aA}	1.348±0.053^{abC}	19.999±0.622^{aA}
	F	26.261***	25.966***	51.441***	53.008***	4.367*	4.786*
C. hystrix	HL	3.083±0.099^{aB}	0.693±0.037^{aB}	0.731±0.086^{bC}	4.508±0.184^{bC}	2.728±0.110^{bAB}	2.989±0.219^{aB}
	ML	2.157±0.103^{bC}	0.457±0.034^{bC}	1.219±0.111^{bC}	3.834±0.173^{bD}	4.175±0.057^{aA}	1.547±0.678^{aB}
	LL	3.384±0.182^{aC}	0.743±0.052^{aC}	2.262±0.238^{aC}	6.389±0.369^{aC}	4.607±0.263^{aA}	2.670±0.473^{aD}
	F	22.946***	13.315***	23.969***	26.240***	34.299***	2.355
B. alnoides	HL	3.850±0.341^{bAB}	0.994±0.103b^{AB}	1.723±0.070^{bB}	6.567±0.447^{bB}	3.386±0.288^{aA}	5.415±0.880^{abB}
	ML	5.662±0.382^{aB}	1.372±0.094^{aB}	1.822±0.076^{bB}	8.856±0.481^{aC}	3.022±0.191^{aB}	3.518±0.524^{bB}
	LL	5.325±0.365^{aB}	1.203±0.059^{abB}	3.356±0.159^{aB}	9.883±0.410^{aB}	2.825±0.163^{aB}	6.289±0.536^{aC}
	F	7.045**	4.658*	70.030***	14.424***	1.665	4.504*
